# Supplementary material for: 3-Ferrocenyl-estra-1,3,5 (10)-triene-17-one: Synthesis, Crystal Structure, Hirshfeld Surface Analysis, DFT Studies, and Its Binding to Human Serum Albumin Studied through Fluorescence Quenching and In Silico Docking Studies
Source: Molecules. 2023 Aug 20;28(16):6147. doi: 10.3390/molecules28166147 (PMC10458889; doi:10.3390/molecules28166147)
Supplement: Supplementary file 1 [file molecules-28-06147-s001.zip › molecules-2561908-supplementary.pdf]

Supplementary Material

3-Ferrocenyl-estra-1,3,5 (10)-triene-17-one: Synthesis, crystal structure, Hirshfeld surface analysis, DFT studies, and its binding to human serum albumin studied by fluorescence quenching and *in silico* docking studies

Mariola M. Flores-Rivera [1], José A. Carmona-Negrón [1], Arnold L. Rheingold [2], and Enrique Meléndez [1,*]

[1] University of Puerto Rico, Department of Chemistry, PO Box 9019, Mayaguez, PR 00681, Puerto Rico.
[2] University of California–San Diego, Department of Chemistry and Biochemistry, Urey Hall 5128, 9500 Gilman Drive, La Jolla, CA 92093-0358, USA.
*Correspondence: enrique.melendez@upr.edu

Table of Contents

1. ^{1}H NMR: 3-ferrocenyl-estra-1,3,5 (10)-triene-17-one 2
2. ^{13}C NMR: 3-ferrocenyl-estra-1,3,5 (10)-triene-17-one 3
3. IR: 3-ferrocenyl-estra-1,3,5 (10)-triene-17-one 4
4. CheckCIF/PLATON report: 3-ferrocenyl-estra-1,3,5 (10)-triene-17-one 5-8
5. In silico docking studies: HSA Site I 9

^{1}H NMR: 3-ferrocenyl-estra-1,3,5 (10)-triene-17-one

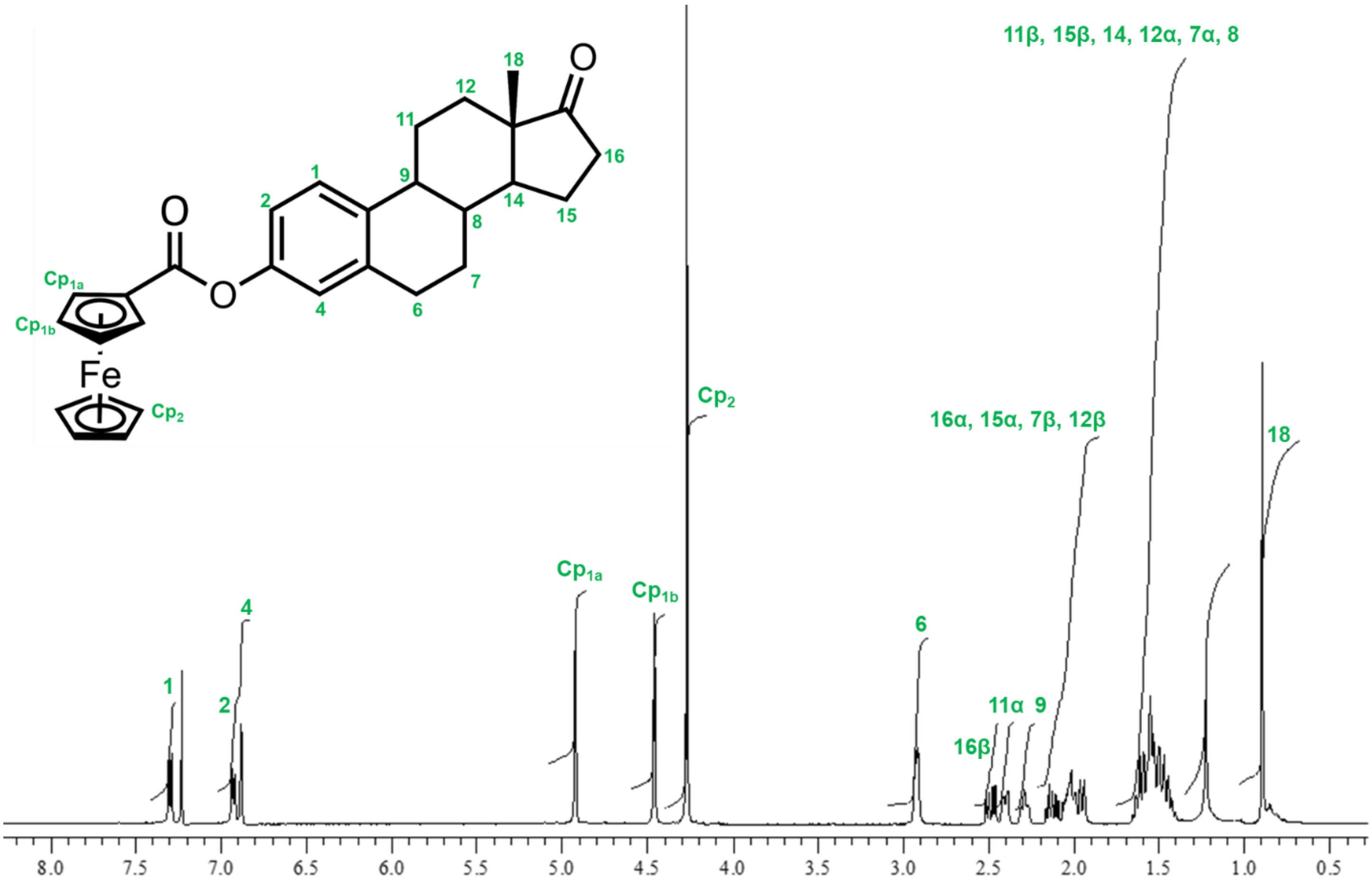

Figure S1: 3-ferrocenyl-estra-1,3,5 (10)-triene-17-one (500 MHz, $CDCl_3$) δ(ppm): 7.34 (d, 1H, ^3J=8.44 Hz; H_1), 6.96(d, 1H, ^3J=8.57 Hz; H_2), 6.92(s, 1H; H_4), 4.96(s, 2H; Cp_{1a}), 4.50(s, 2H; Cp_{1b}), 4.31(s, 5H; Cp_2), 2.96(m, 2H; H_6), 2.53(dd, ^3J=18.92,1H, ^3J=8.69; $H_{16β}$), 2.44(m, 1H; $H_{11α}$), 2.33(m, 1H; H_9), 0.94(s, 3H; H_{18a}, H_{18b}, and H_{18c}).

^{13}C NMR: 3-ferrocenyl-estra-1,3,5 (10)-triene-17-one

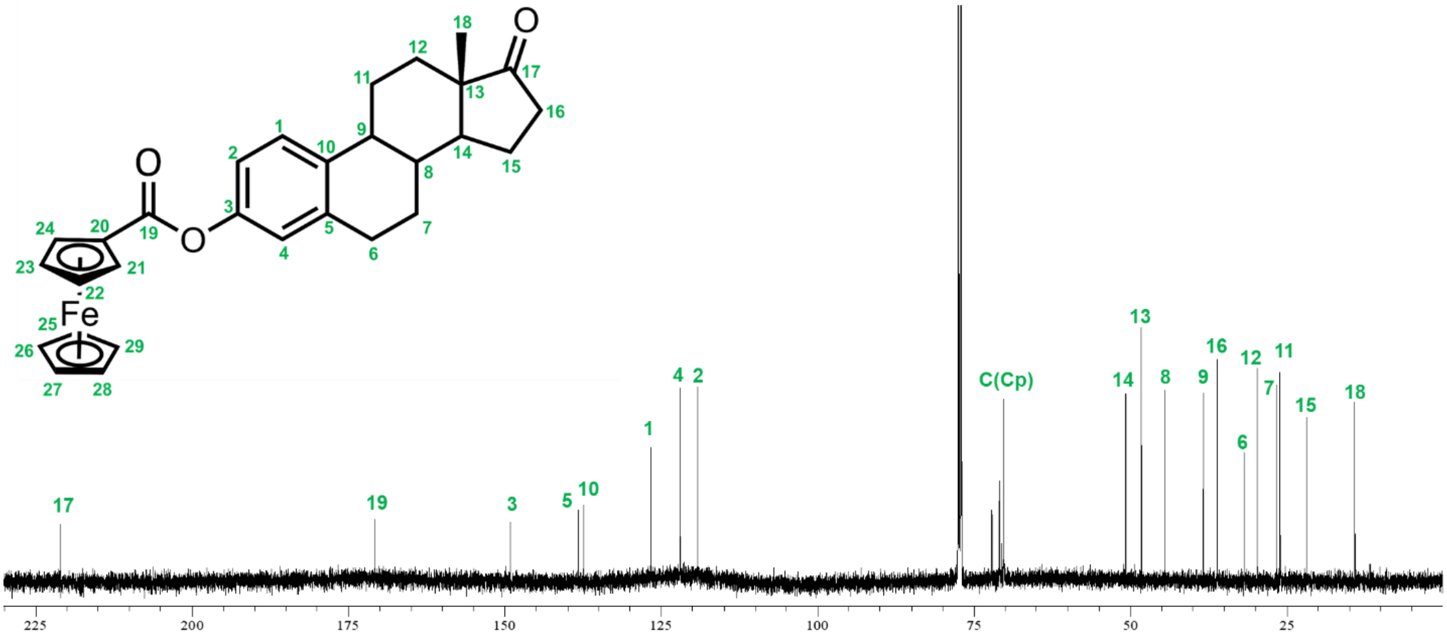

Figure S2: 3-ferrocenyl-estra-1,3,5 (10)-triene-17-one ^{13}C NMR (125 MHz, $CDCl_3$), δ(ppm): 220.9 (C_{17}), 170.77 (C_{19}), 149.01 (C_3), 138.23 (C_5), 137.31 (C_{10}), 126.61 (C_1), 121.91 (C_4), 119.12 (C_2), 72.10 (Cp - C_{20}), 70.92 (Cp - C_{21} and C_{24}), 70.51 (Cp - C_{22} and C_{23}), 70.23 (Cp - C_{25}, C_{26}, C_{27}, C_{28} d C_{29}), 50.78 (C_{14}), 48.21 (C_{13}), 44.42 (C_8), 38.33 (C_9), 36.61 (C_{16}), 31.82 (C_{12}), 29.76 (C_6), 26.76 (C_7), 26.00 (C_{11}), 21.80 (C_{15}), 14.10 (C_{18}).

IR: 3-ferrocenyl-estra-1,3,5 (10)-triene-17-one

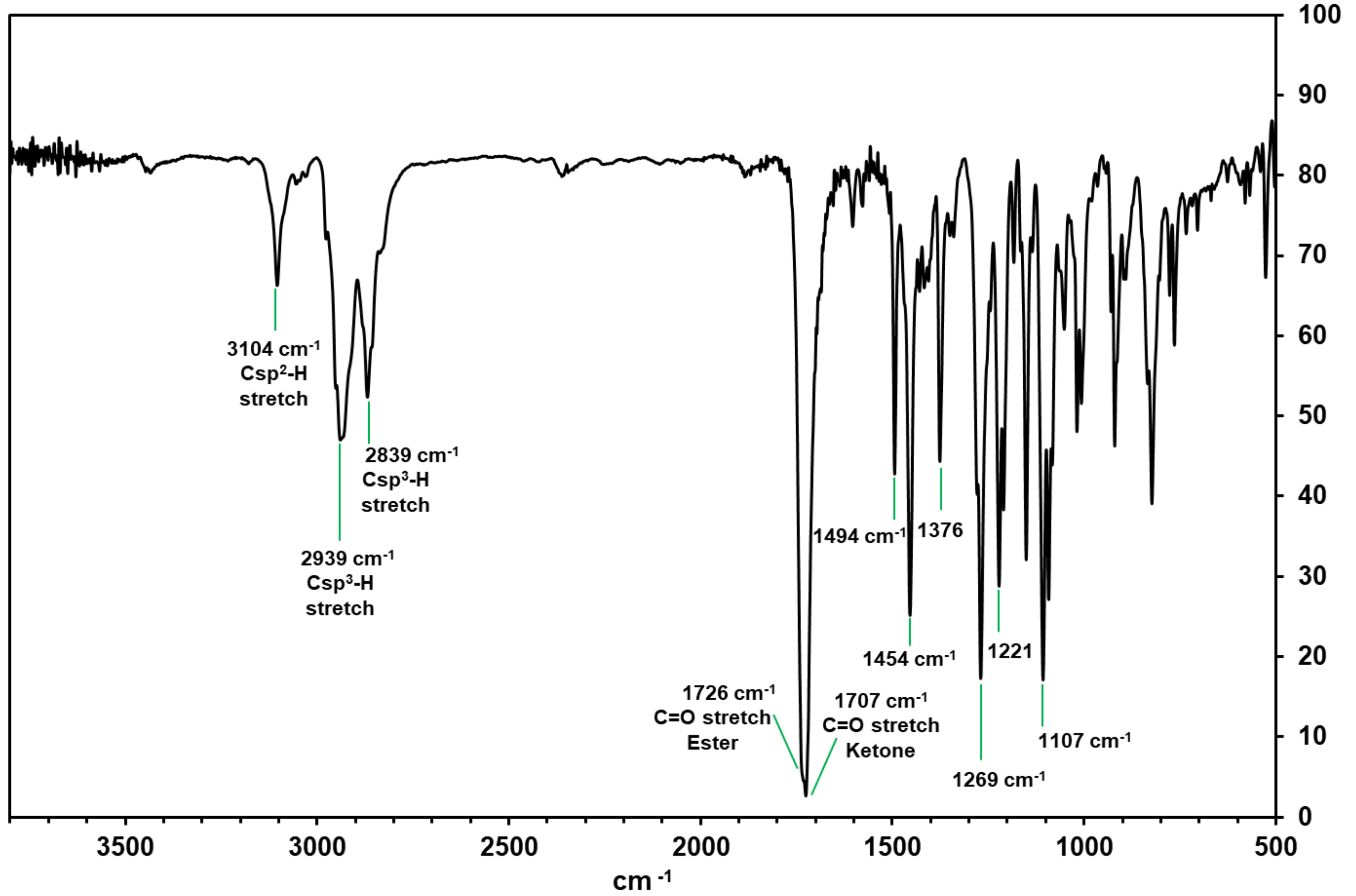

Figure S3: IR spectra for 3-ferrocenyl-estra-1,3,5 (10)-triene-17-one (KBr, cm^{-1}): 3104, 2939, 2869, 1726, 1707, 1494, 1454, 1376, 1269, 1221, 1107.

checkCIF/PLATON report

You have not supplied any structure factors. As a result the full set of tests cannot be run.

THIS REPORT IS FOR GUIDANCE ONLY. IF USED AS PART OF A REVIEW PROCEDURE FOR PUBLICATION, IT SHOULD NOT REPLACE THE EXPERTISE OF AN EXPERIENCED CRYSTALLOGRAPHIC REFEREE.

No syntax errors found. CIF dictionary Interpreting this report

Datablock: emel25r_0m_a

```
Bond precision:   C-C = 0.0031 A            Wavelength=0.71073

Cell:            a=10.5771(6)     b=11.1471(7)        c=20.0257(12)
                 alpha=90         beta=102.174(1)     gamma=90
Temperature:     100 K
```

	Calculated	Reported
Volume	2308.0(2)	2308.0(2)
Space group	C 2	C 1 2 1
Hall group	C 2y	C 2y
Moiety formula	C29 H30 Fe O3	C29 H30 Fe O3
Sum formula	C29 H30 Fe O3	C29 H30 Fe O3
Mr	482.38	482.38
Dx,g cm-3	1.388	1.388
Z	4	4
Mu (mm-1)	0.683	0.683
F000	1016.0	1016.0
F000’	1017.76	
h,k,lmax	14,14,26	14,14,26
Nref	5781[3036]	5778
Tmin,Tmax	0.826,0.849	0.695,0.746
Tmin’	0.826	

```
Correction method= # Reported T Limits: Tmin=0.695 Tmax=0.746
AbsCorr = MULTI-SCAN

Data completeness= 1.90/1.00        Theta(max)= 28.350

R(reflections)= 0.0247( 5568)          wR2(reflections)= 0.0619( 5778)

S = 1.032                   Npar= 299
```

The following ALERTS were generated. Each ALERT has the format
test-name_ALERT_alert-type_alert-level.
Click on the hyperlinks for more details of the test.

Alert level C

```
PLAT213_ALERT_2_C Atom O3              has ADP max/min Ratio .....        3.1 prolat
```

Alert level G

```
PLAT005_ALERT_5_G No Embedded Refinement Details Found  in the CIF     Please Do !
PLAT794_ALERT_5_G Tentative Bond Valency for Fe1        (II)      .       2.14 Info
```

```
   0 ALERT level A = Most likely a serious problem - resolve or explain
   0 ALERT level B = A potentially serious problem, consider carefully
   1 ALERT level C = Check. Ensure it is not caused by an omission or oversight
   2 ALERT level G = General information/check it is not something unexpected

   0 ALERT type 1 CIF construction/syntax error, inconsistent or missing data
   1 ALERT type 2 Indicator that the structure model may be wrong or deficient
   0 ALERT type 3 Indicator that the structure quality may be low
   0 ALERT type 4 Improvement, methodology, query or suggestion
   2 ALERT type 5 Informative message, check
```

Validation response form

Please find below a validation response form (VRF) that can be filled in and pasted into your CIF.

```
# start Validation Reply Form
_vrf_PLAT213_emel25r_0m_a
;
PROBLEM: Atom O3              has ADP max/min Ratio .....        3.1 prolat
RESPONSE: ...
;
# end Validation Reply Form
```

It is advisable to attempt to resolve as many as possible of the alerts in all categories. Often the minor alerts point to easily fixed oversights, errors and omissions in your CIF or refinement strategy, so attention to these fine details can be worthwhile. In order to resolve some of the more serious problems it may be necessary to carry out additional measurements or structure refinements. However, the purpose of your study may justify the reported deviations and the more serious of these should normally be commented upon in the discussion or experimental section of a paper or in the "special_details" fields of the CIF. checkCIF was carefully designed to identify outliers and unusual parameters, but every test has its limitations and alerts that are not important in a particular case may appear. Conversely, the absence of alerts does not guarantee there are no aspects of the results needing attention. It is up to the individual to critically assess their own results and, if necessary, seek expert advice.

Publication of your CIF in IUCr journals

A basic structural check has been run on your CIF. These basic checks will be run on all CIFs submitted for publication in IUCr journals (*Acta Crystallographica*, *Journal of Applied Crystallography*, *Journal of Synchrotron Radiation*); however, if you intend to submit to *Acta Crystallographica Section C* or *E* or *IUCrData*, you should make sure that full publication checks are run on the final version of your CIF prior to submission.

Publication of your CIF in other journals

Please refer to the *Notes for Authors* of the relevant journal for any special instructions relating to CIF submission.

PLATON version of 06/07/2023; check.def file version of 30/06/2023

Datablock emel25r_0m_a - ellipsoid plot

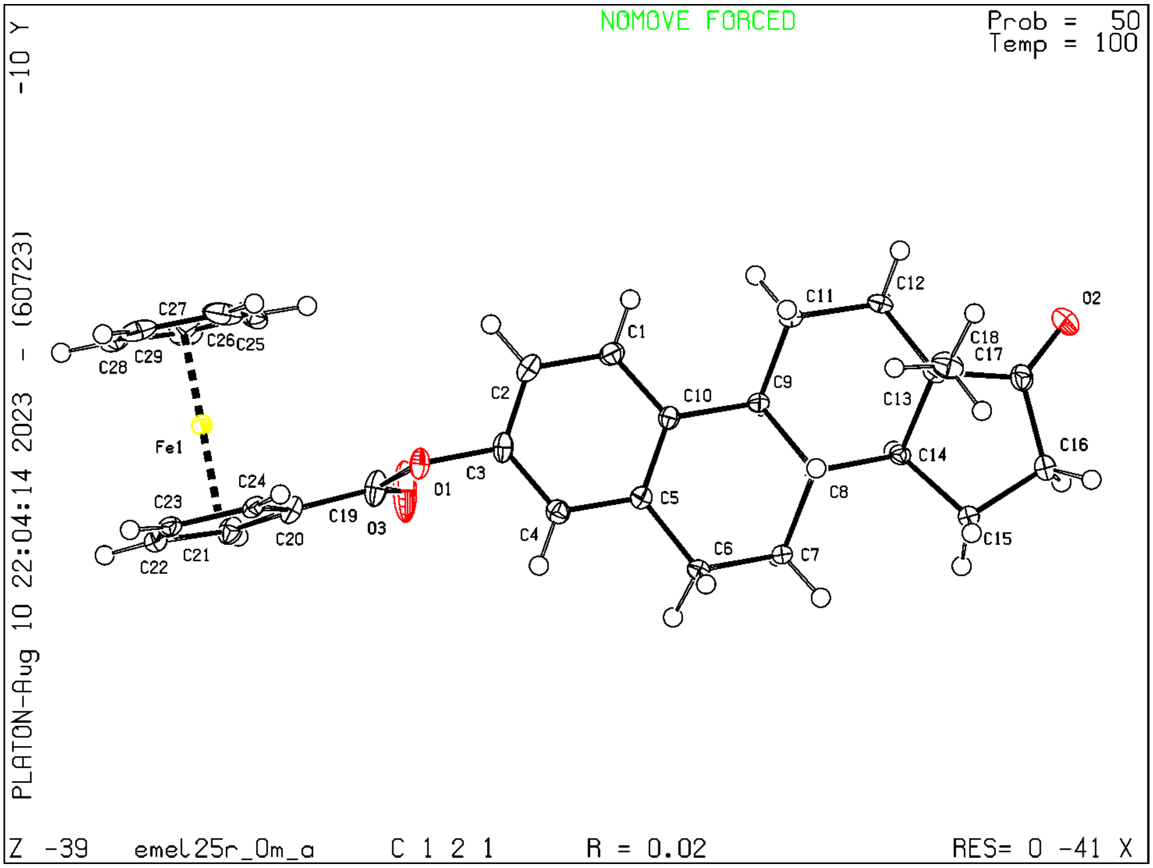

In silico docking studies: HSA Site I

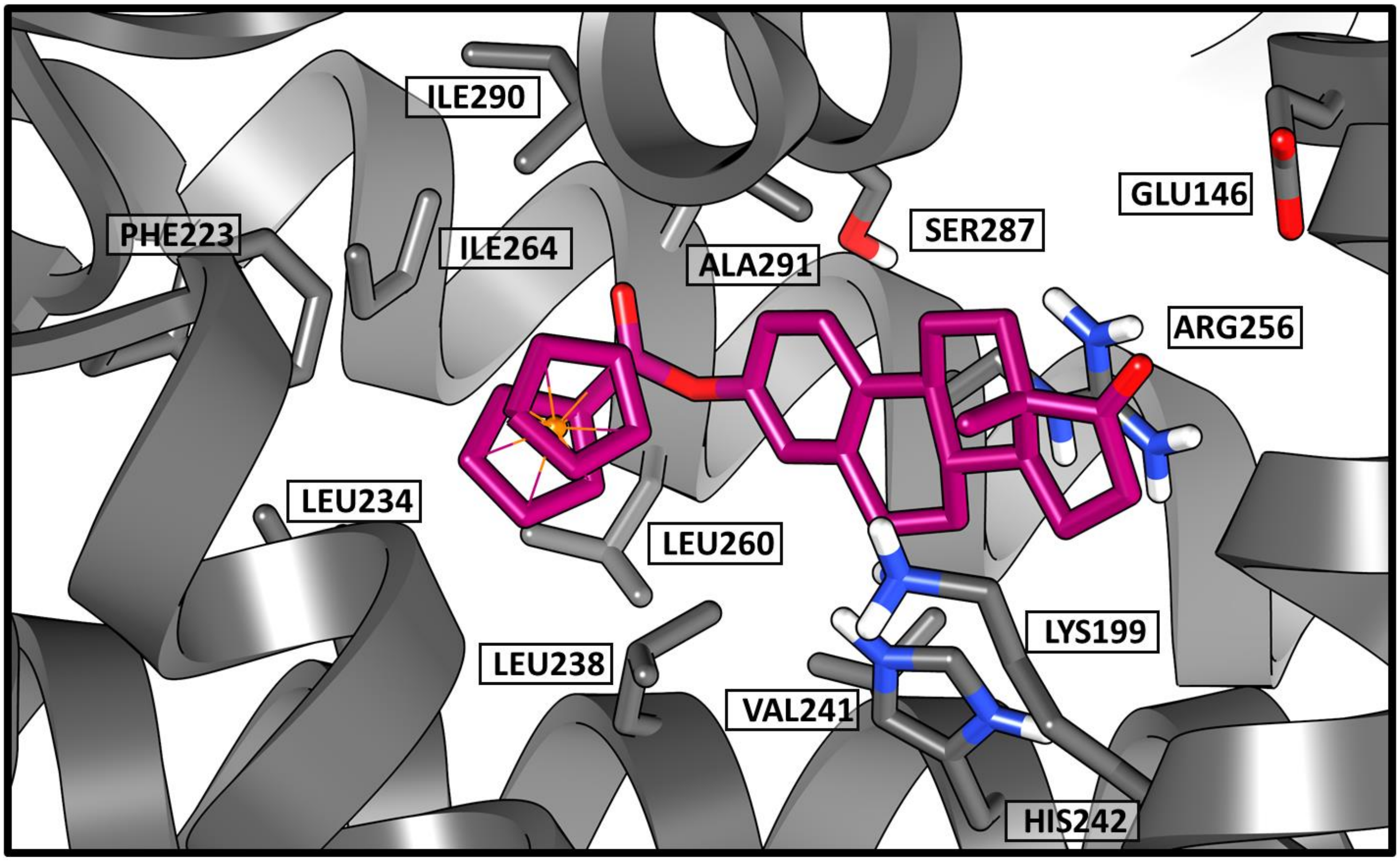

Figure S4: Docking pose of 3-ferrocenyl-estra-1,3,5 (10)-triene-17-one (2) inside the human serum albumin (HSA) drug-binding site I.
